# Impact of environmental and socioeconomic factors on the prevalence of and DALYs due to cutaneous leishmaniasis globally from 1990 to 2021 based on remote sensing and GIS technologies

**DOI:** 10.1186/s13071-026-07395-0

**Published:** 2026-04-15

**Authors:** Xinyi Chen, Bin Xie, Yinan Cui, Hanlu Li, Xingyun Wang, Jiahao Zhou, Zhe Lu

**Affiliations:** 1 School of Basic Medical Sciences, Hangzhou Normal University, 2318th Yuhangtang Road, Yuhang District, Hangzhou, 311121 China; 2 School of Information Science and Technology, Hangzhou Normal University, Hangzhou, 311121 China; 3 Kharkiv Institute at Hangzhou Normal University, Hangzhou Normal University, Hangzhou, 311121 China; 4 Zhejiang Key Laboratory of Medical Epigenetics, Hangzhou Normal University, Hangzhou, 311121 China; 5 Zhejiang Provincial Key Laboratory of Wetland Intelligent Monitoring and Ecological Restoration, Hangzhou Normal University, Hangzhou, 311121 China; 6 The Center for Caribbean Studies at Hangzhou Normal University, Hangzhou Normal University, Hangzhou, 311121 China

**Keywords:** Leishmaniasis, Geographic information system, Influencing factors, Spatiotemporal clustering, GTWR

## Abstract

**Background:**

Leishmaniasis, a parasitic disease caused by *Leishmania* spp., is a major public health threat. The synergistic effects of environmental and socioeconomic factors on the global distribution of leishmaniasis are unknown.

**Methods:**

Applying epidemiological data on cutaneous leishmaniasis (CL) from the Global Burden of Disease 2021 database, we used spatial autocorrelation and standard deviation ellipses to explore the spatiotemporal clustering and migration patterns of CL. Four remote sensing-retrieved environmental factors and five socioeconomic factors were selected for analysis. Spearman’s correlation coefficient was used to screen for factors correlated with the prevalence of and disability-adjusted life years (DALYs) due to CL. Ordinary least squares (OLS), geographically weighted regression (GWR) and geographically and temporally weighted regression (GTWR) were used to assess the impact of the influencing factors on the prevalence of and DALYs due to CL.

**Results:**

From 1990 to 2008, the global prevalence of and DALYs due to CL exhibited significant positive spatial autocorrelation (*Z* > 1.96, *P* < 0.05). Prevalence and DALYs both had one cold spot, located in northern Africa, and two hot spots, located in Central America and Central Asia. Temperature, infant mortality rate (IMR) and humidity were significantly positively correlated with the prevalence of and DALYs due to CL, whereas gross domestic product (GDP) and surface solar radiation (SSR) were significantly negatively correlated with the latter. The GTWR model demonstrated the best regression performance, with adjusted *R*^2^ values for prevalence reaching 0.841, 0.984, 0.839 and 0.972, and those for DALYs reaching 0.816, 0.966, 0.837 and 0.972 in Asia, Europe, the Americas and Africa, respectively. Regression coefficients further quantified the individual contributions of each factor to the prevalence of and DALYs due to CL, which could provide a scientific basis for governments to implement targeted control of CL.

**Conclusions:**

To our knowledge, this study is the first to analyze the global spatiotemporal distribution patterns of the prevalence of and DALYs due to CL and quantitatively study the spatiotemporal effects of environmental and socioeconomic factors on CL on a global scale. Environmental (temperature, SSR and humidity) and socioeconomic (GDP and IMR) factors were significantly correlated with the prevalence of and DALYs due to CL. The GTWR model outperformed the GWR and OLS models, further confirming the spatiotemporal effects of influencing factors on CL.

**Graphical Abstract:**

**Figure:**
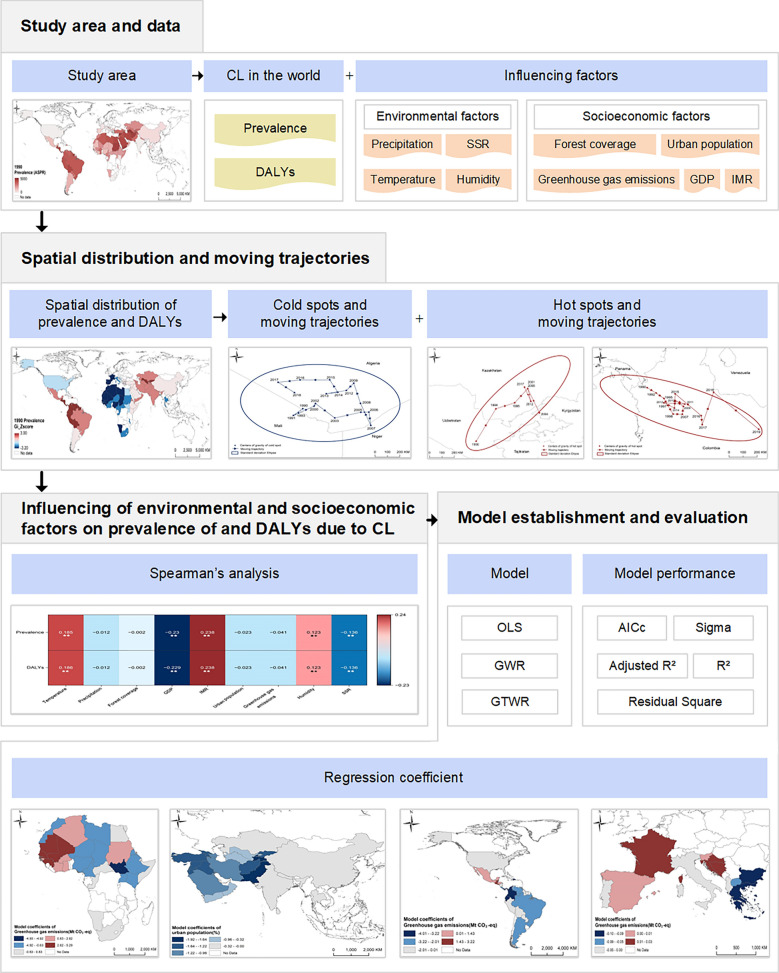

## Background

Leishmaniasis is a tropical disease caused by > 20 species of *Leishmania* protozoa. Based on the heterogeneity of its clinical manifestations, it can be classified into cutaneous leishmaniasis (CL), mucocutaneous leishmaniasis (MCL) and visceral leishmaniasis (VL) [1], whereby MCL is generally classified as a clinical form of CL and thus included in the CL category [2]. In this study, CL refers to the cases of both CL and ML.

Leishmaniasis is prevalent in 98 countries worldwide [3]. In 2023, the WHO reported 272,098 and 11,922 new cases of CL and VL, respectively [4]. Leishmaniasis is a major health issue in the Americas, East Africa, North Africa, West Asia and Southeast Asia. From 1990 to 2021, the number of CL cases showed a fluctuating upward trend [5], whereas the number of VL cases showed an overall downward trend [6]. Undeveloped surveillance systems in remote areas, insufficient diagnostic abilities and clinical confusion with other skin diseases have influenced CL treatment [7]. Early skin lesions are often incorrectly diagnosed as bacterial infections such as tuberculosis, leading to delayed treatment.

Epidemiological studies of leishmaniasis require careful consideration of its spatial and temporal heterogeneity. Sand flies are the intermediate hosts of *Leishmania*, whose primary mode of infection is through the bites of female sand flies during blood-feeding, and sand fly activity shows clear regional characteristics. For example, in Gansu Province, China, VL cases are mainly distributed along the Yellow River and its tributaries (such as the Tao River and Wei River), with distinct clustering along water systems [8]. In the Mediterranean region, cases peak from summer to early autumn, coinciding with the reproductive season of sand flies [9], while in northwestern China, influenced by a continental climate, transmission peaks from June to August [10]. Human activities can also affect the occurrence of CL. In French Guiana, 30 infectious cases were confirmed among soldiers following nighttime combat training in a rainforest base in mid-May 2020, resulting in an atypically small peak of infection [11]. These differences indicate that geographical (such as climate and hydrology) and social factors collectively shape disease transmission patterns. However, to date, no study has quantitatively analyzed the distributional patterns of CL or explored the associated environmental, social and economic factors on a global scale.

Geographic information system (GIS) and remote sensing (RS) technologies are important disease investigation tools. RS technologies capture full-coverage and high-resolution environmental data, such as vegetation indices and land-use types, providing important environmental data for modeling [12]. GIS utilizes spatial analysis and visualization functions to generate disease risk maps, facilitating the understanding of future risk trends. Abdol et al. mapped the prevalence of VL in Iran using the ArcMap geospatial processing program and found a nonlinear regression relationship between environmental factors (vegetation and elevation) and the prevalence of VL [13]. A study in Central Iran determined the spatial distribution of CL incidence using GIS and identified high-risk points in Isfahan Province using Moran’s index (Moran’s I); however, the authors did not carry out further investigations into the potential influence of natural environmental and socioeconomic factors [14]. Similarly, based on national surveillance datasets in Sri Lanka from 2001 to 2018, the Getis–Ord Gi* spatial analysis tool was used to identify domestic hot spots of CL and analyze their spatiotemporal evolution characteristics; however, again the underlying influencing factors were not investigated [15]. Taken together, currently available research is confined to the national or regional level and lacks global-scale investigation.

Ordinary least squares (OLS), which assumes that relationships between variables are consistent without considering spatiotemporal heterogeneity, geographically weighted regression (GWR), which posits that relationships between variables are spatially nonstationary, with the regression coefficients being local parameters determined by geographic location, and geographically and temporally weighted regression (GTWR), which advances the GWR framework by accounting for temporal nonstationarity, thereby generating a unique set of local parameters for each spatiotemporal point, are widely used in disease research. These statistical methods have been used in epidemiological studies. Kianfar et al. [16] used Global Moran’s I and Getis–Ord Gi* to examine the spatiotemporal distribution patterns and high-risk areas of COVID-19 in Europe, respectively. In addition, these authors compiled 40 potential explanatory variables and used multiple models, including OLS and GWR, to investigate the spatial and temporal variations in COVID-19 cumulative incidence rates. In their study on dengue hemorrhagic fever in Indonesia, Herdianti et al. [17] used GWR to explore the spatial impact of larval mosquito density and living environment on the incidence of dengue fever. Yu et al. [18] used OLS, GWR and GTWR to study the spatiotemporal distribution changes in the incidence of pulmonary tuberculosis in China from 2004 to 2021. In the field of cancer research, Roquette et al. [19] used GWR to compare the spatial distribution patterns of the incidence and mortality of colorectal cancer in Portugal. Guo et al. [20] used GTWR to analyze factors influencing the incidence of lung cancer in China. Xie et al. [21] examined the effects of 20 socio-environmental factors on the incidence and mortality of lung cancer in China between 2007 and 2016 using OLS, GWR and GTWR. Although these models have been widely applied in disease research, their application globally to explain factors influencing CL has received negligible attention.

Based on RS-retrieved data and GIS, this study reveals the synergistic effects of environmental and socioeconomic factors on CL globally with the specific aims to: (i) explore the spatial clustering characteristics of the prevalence of and disability-adjusted life years (DALYs) due to CL over 32 years (1990–2021); (ii) monitor the moving trajectories of the centers of gravity of the cold and hot spots of the prevalence of and DALYs due to CL; (iii) determine the correlation between various environmental and socioeconomic factors and the prevalence of and DALYs due to CL using Spearman’s correlation analysis; and (iv) reveal the relationship between the correlated environmental and socioeconomic factors and the prevalence of and DALYs due to CL based on OLS, GWR and GTWR models.

**Fig. 1:**
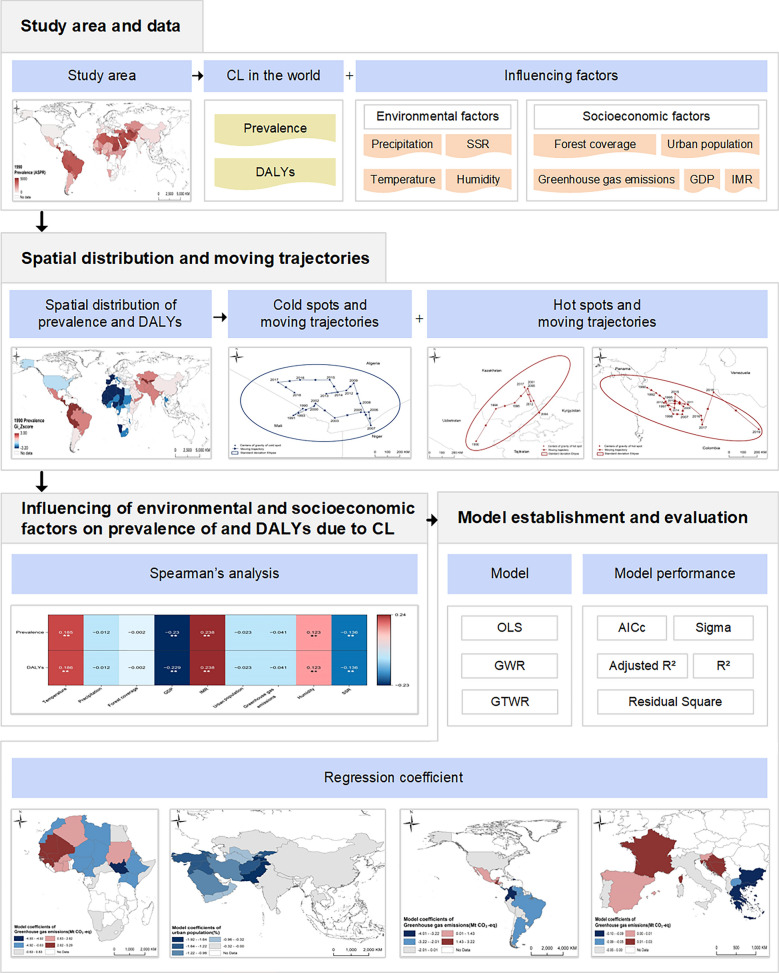
Workflow of the study. AICc, Akaike information criterion corrected; CL, cutaneous leishmaniasis; DALYs, disability-adjusted life years; GDP, gross domestic product; GTWR, geographically and temporally weighted regression; GWR, geographically weighted regression; IMR, infant mortality rate; OLS, ordinary least squares; R^2^, coefficient of determination.

The workflow of this study is illustrated in Fig. 1. Our study is the first to reveal the cold and hot spots of CL and their spatiotemporal changes from 1990 to 2021 and to explore the factors influencing these on a global scale. These findings may provide a scientific basis for controlling risk factors, thereby reducing the burden of CL.

## Methods

### Study area

This study included 92 countries with complete epidemiological data on CL from 1990 to 2021 in the Global Burden of Disease (GBD) 2021 database (https://ghdx.healthdata.org/gbd-2021), spanning Asia, Europe, the Americas and Africa.

### Data collection and preparation

This study used a world map with the WGS1984 geographic coordinate system. The collected data included the prevalence of and DALYs due to CL (International Classification of Diseases [ICD] code: B55.1) from the GBD database and nine environmental and socioeconomic factors. All data sources are listed in Table 1.

**Table 1:** Data sources

Data type	Indicator	Duration	Source
Cutaneous leishmaniasis	Prevalence	1990–2021	GBD database: https://vizhub.healthdata.org/gbd-results/ Accessed on 18 June 2024
	DALYs		
Environmental factors	Precipitation	1990/1995/	Climate Change Knowledge Portal: https://climateknowledgeportal.worldbank.org/download-data Accessed on 27 July 2024
	Temperature	2000/2005/2010/2015/2020	
	Humidity	1990/1995/2000/2005/2010/2015/2020	Climate Data Store: https://cds.climate.copernicus.eu/datasets/derived-near-surface-meteorological-variables?tab=overview Accessed on 10 September 2024
	SSR	1990/1995/2000/2005/2010/2015	Global Integrated and Uniform Solar Surface Radiation Dataset (1955–2018) provided by National Cryosphere Desert Data Center: https://www.ncdc.ac.cn/portal/metadata/c09739c5-9f1c-4f8a-9bfa-5ec83e90299e Accessed on 23 July 2024
		2015/2020	National Tibetan Plateau Data Center: https://data.tpdc.ac.cn/zh-hans/data/bae0f399-00bc-43f0-8fa6-501892392f98 Accessed on 29 July 2024
Socioeconomic factors	Forest coverage	1990/1995/	World Bank Open Data: https://data.worldbank.org.cn/ Accessed on 19 September 2024
	GDP	2000/2005/	
	IMR	2010/2015/	
	Urban population	2020	
	Greenhouse gas emissions		

DALYs, disability-adjusted life years; GBD, Global Burden of Disease; GDP, gross domestic product; IMR, infant mortality rate.

#### Epidemiological data on CL

**Fig. 2:**
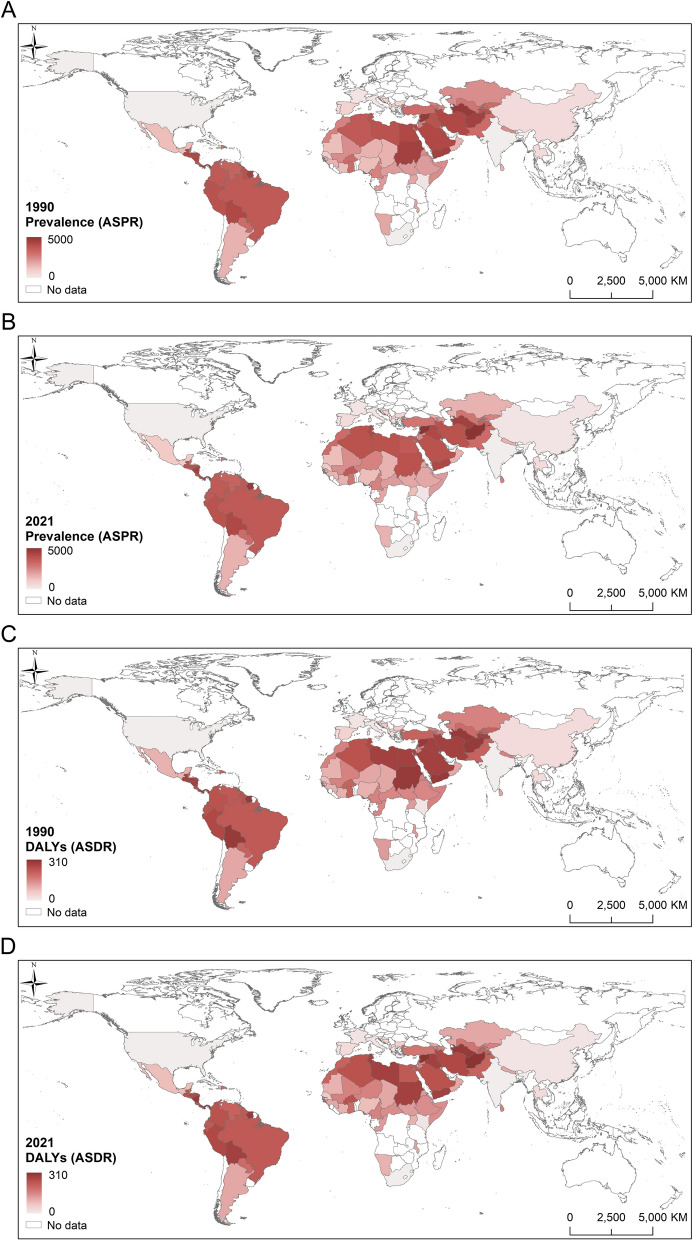
The prevalence of and DALYs due to cutaneous leishmaniasis (CL) in 1990 and 2021. **A, B** Prevalence of CL in 1990 (**A**) and in 2021 (**B**). **C, D** DALYs due to CL in 1990 (**C**) and in 2021 (**D**). ASDR, Age-standardized DALYs rate; ASPR, age-standardized prevalence rate, DALYs, disability-adjusted life years.

Epidemiological data, including the age-standardized prevalence rate (ASPR) and age-standardized DALYs rate (ASDR) of CL, were obtained from the GBD maintained by the Institute for Health Metrics and Evaluation at the University of Washington (USA). To ensure the integrity of the study, long-term statistical data from 1990 to 2021 in GBD 2021 (http://ghdx.healthdata.org/gbd-results-tool) were downloaded for analysis. The prevalence of and DALYs due to CL in 1990 and 2021 were plotted in Fig. 2.

#### Precipitation and surface temperature

The annual average precipitation and surface temperatures for 92 countries every 5 years from 1990 to 2020 were obtained from the World Bank's Climate Change Knowledge Portal website (https://climateknowledgeportal.worldbank.org/) and the Climatic Research Unit Time Series (CRU TS) global dataset covering land areas (https://worldbank.github.io/climateknowledgeportal/docs/collections/cru-x0.5.html).

#### Humidity

Humidity data for 92 countries were collected every 5 years from 1990 to 2021 from the Copernicus Climate Change Service (https://climate.copernicus.eu/). This dataset was generated using the WATCH Forcing Data methodology applied to ERA5, making it suitable for long-term series climate research and high-precision climate simulations.

We used ArcGIS (ArcMAP 10.8) to batch-convert the monthly data to a Tag Image File Format and used the Raster Calculator tool to obtain annual raster data. Finally, based on the administrative division of the data, we assigned raster data to the shapefiles of countries worldwide and converted them into national-level data.

#### Surface solar radiation

Surface solar radiation (SSR) data were derived from a fused global land surface longwave downward radiation dataset from the National Tibetan Plateau/Third Pole Environment Data Center (Tibet) and from a global integrated and uniform SSR dataset from the National Cryosphere Desert Data Center (China).

The global integrated and uniform SSR dataset covers the period 1955–2018, and the fused global land surface longwave downward radiation dataset covers the period 2000–2020. Neither dataset can directly or completely cover the 1990–2020 period. Therefore, a regression analysis was conducted using global integrated and uniform SSR data from 2015 as the independent variable and fused global land surface longwave downward radiation data from 2015 as the dependent variable, using IBM SPSS Statistics 27 (IBM Corp., Armonk, NY, USA). The global integrated and uniform SSR dataset was obtained as monthly averages that were then converted into annual averages. Finally, the fused global land surface longwave downward radiation dataset from 2020, which was converted into the same format as the global integrated and uniform SSR data, was used along with the global integrated and uniform SSR data from 1990/1995/2000/2005/2010/2015.

#### Socioeconomic factors

Data on forest coverage, gross domestic product (GDP), infant mortality rate (IMR), urban population and greenhouse gas emissions every 5 years from 1990 to 2020 were obtained from World Bank Open Data (https://data.worldbank.org.cn/). These data originate from official statistical agencies, international organizations (e.g., United Nations and Food and Agriculture Organization) and national governments, and are annual data at the national level.

### Methods

#### Spatial clustering analysis

Spatial autocorrelation has revealed correlations between geographically adjacent locations [22] and is an important tool for analyzing the geographic heterogeneity of spatial data. In this study, Global Moran’s I and Local Getis–Ord Gi* statistics [23, 24], available in the spatial autocorrelation and hot spot analysis toolbox of ArcGIS (ArcMAP 10.8 application; ESRI [Environmental Systems Research Institute], Redlands, CA, USA), respectively, were used to investigate the spatial clustering characteristics of CL and identify cold and hot spots. In addition, a standard deviation ellipse analysis was employed to reveal the moving trajectories of the centers of gravity of the cold and hot spots of the CL.

(i) Global Moran's ***I***

Global Moran’s I [24] is a commonly used indicator of global spatial autocorrelation, which assesses whether spatial clustering exists across a study area. The formula used is as follows [25]:

1 $$I = \frac{{n\mathop \sum \nolimits_{i = 1}^{n} \mathop \sum \nolimits_{j = 1}^{n} w_{ij} (y_{i} - \overline{y})\left( {y_{j} - \overline{y}} \right)}}{{s_{0} \mathop \sum \nolimits_{i = 1}^{n} \left( {y_{i} - \overline{y}} \right)^{2} }}$$,

 where $n$ represents the total sample size of spatial units within the study area; ${y}_{i}$ and ${y}_{j}$ are the attribute values of spatial units $i$ and $j$, respectively; $\overline{y }$ is the mean of all attribute values; ${w}_{ij}$ is the spatial weight between units $i$ and $j$; and ${s}_{0}={\sum }_{i=1}^{n}\sum_{j=1}^{n}{w}_{ij}$ is the sum of all elements in the weight matrix. The Global Moran’s *I* is in the range [− 1, 1]. The larger the absolute value, the stronger the spatial autocorrelation; zero represents a random distribution.

(ii) Getis–Ord Gi*

The Getis–Ord Gi* statistic was used to identify statistically significant spatial clusters with high and low values. It measures the standardized difference between the local and global means. The formula used is as follows [23]:

2 $$G_{i}^{*} = \frac{{\mathop \sum \nolimits_{j = 1}^{n} w_{ij} x_{j} - \overline{x}\mathop \sum \nolimits_{j = 1}^{n} w_{ij} }}{{S\sqrt {\frac{{n\mathop \sum \nolimits_{j = 1}^{n} w_{ij}^{2} - \left( {\mathop \sum \nolimits_{j = 1}^{n} w_{ij} } \right)^{2} }}{n - 1}} }}$$,

 where *i* denotes the central element, *j* denotes all elements in its neighborhood; ${x}_{j}$ is the disease incidence in region *j*; $\overline{x}$ is the global mean incidence; ${w}_{ij}$ is the spatial weight; *s* is the standard deviation; and *n* is the count of all neighboring elements.

A Z-test and the corresponding *P* values were used to assess the statistical significance of Gi* values, with significance levels typically set at 0.05 or 0.01. Significantly hot (Gi* values are significantly positive) and cold (Gi* values are significantly negative) spots were identified, and their geographical features and potential influencing factors were analyzed.

(iii) Standard deviation ellipse

The standard deviation ellipse visually represents the spatial distribution of the centers of gravity of the hot and cold spots, revealing their directional biases and dispersion trends. The latitude and longitude coordinates were converted into a projected coordinate system, and a standard deviation ellipse was fitted to the case points using the least squares method to reflect the spatial movement trends and diffusion patterns. The center of gravity is calculated as follows [26]:

3 $$(\overline{X},\overline{Y}) = \frac{{\sum\nolimits_{i = 1}^{n} {M_{i} } X_{i} }}{{\sum\nolimits_{i = 1}^{n} {M_{i} } }},\frac{{\sum\nolimits_{i = 1}^{n} {M_{i} } Y_{i} }}{{\sum\nolimits_{i = 1}^{n} {M_{i} } }}$$,

 where $\stackrel{-}{(X},\overline{Y })$ are the coordinates of the center of gravity; *n* is the count of all points; *M*_*i*_ is the weight of point *i*; and *X*_*i*_ and *Y*_*i*_ are the longitude and latitude of point *i*, respectively.

#### Spearman’s correlation coefficient

Spearman’s correlation coefficient is a nonparametric measure used to assess the strength and direction of the association between two ranked variables, particularly when the data do not follow a normal distribution or exhibit nonlinear relationships [27]. It is calculated based on the difference in ranks between paired observations as follows:

4 $$\rho =\frac{{\sum }_{i}({x}_{i}-\overline{x })({y}_{i}-\overline{y })}{\sqrt{{\sum }_{i}{({x}_{i}-\overline{x })}^{2}{\sum }_{i}{({y}_{i}-\overline{y })}^{2}}}$$,

 where ${x}_{i}$ and ${y}_{i}$ represent the ranks derived from the original data transformation; $\overline{x }$ and $\overline{y }$ denote the arithmetic means of the variables *x* and *y*, respectively; ${\sum }_{i}({x}_{i}-\overline{x })({y}_{i}-\overline{y })$ represents the sum of products of deviations, which reflects the degree of monotonic synchronicity between the two variables. The denominator, $\sqrt{{\sum }_{i}{({x}_{i}-\overline{x })}^{2}{\sum }_{i}{({y}_{i}-\overline{y })}^{2}}$, serves to normalize the coefficient $\rho$, constraining its value within the interval of [− 1, 1]. A value of 1 indicates a perfectly positive correlation, − 1 indicates a perfectly negative correlation and 0 indicates no monotonic relationship. The closer the absolute value of *ρ* is to 1, the stronger the monotonic relationship.

#### Spatial statistical models

An OLS regression model [28, 29] was established with the prevalence of or DALYs due to CL as the dependent variable *Y* and the selected environmental and socioeconomic factors ${X}_{i}$ as independent variables. The model is expressed as [30]:

5 $$Y={\beta }_{0}+\sum_{i=1}^{k}{\beta }_{i}{X}_{i}+\epsilon$$,

where $Y$ is the dependent variable; ${X}_{i}$ represents the *i*th independent variable; ${\beta }_{0}$ represents the overall expected level of $Y$ when all ${X}_{i}$ are simultaneously equal to 0; ${\beta }_{i}$ is the regression coefficient; *k* is the number of factors; and $\epsilon$ is the error.

The GWR model allows the regression coefficients to vary spatially, reflecting the local influence of factors on the prevalence or DALYs of the disease. The model is expressed as [31]:

6 $${Y}_{i}={\beta }_{0}({u}_{i},{v}_{i})+\sum_{j=1}^{k}{\beta }_{j}{({u}_{i},{v}_{i}){X}_{ij}+\epsilon }_{i}$$,

where ${Y}_{i}$ represents the observed value of the dependent variable for the *i*th spatial sample; ${X}_{ij}$ represents the *j*th independent variable measured at the *i*th sample point; ${\beta }_{0}({u}_{i},{v}_{i})$ is the local intercept, which varies spatially and reflects the inherent differences in each location; $({u}_{i},{v}_{i})$ are the geographic coordinates of location *i*; ${\beta }_{j}({u}_{i},{v}_{i})$ is the local regression coefficient for variable ${X}_{ij}$; and ${\epsilon }_{i}$ represents the spatial residual between the observed value of ${Y}_{i}$ and its locally fitted value at location $({u}_{i},{v}_{i})$ based on the location-specific coefficients.

The GTWR model allows regression coefficients to vary spatially and temporally, making it a suitable model for analyzing the spatiotemporal dynamics of disease risk. The model is expressed as [32]:

7 $${Y}_{it}={\beta }_{0}({u}_{i},{v}_{i},{t}_{i})+\sum_{j=1}^{k}{\beta }_{j}{({u}_{i},{v}_{i},{t}_{i}){X}_{itj}+\epsilon }_{it}$$,

where ${Y}_{it}$ is the observed value of the dependent variable for spatial unit *i* at time *t*; ${X}_{itj}$ denotes the observed value of the *j*th explanatory variable at location *i* and time *t*; ${\beta }_{0}({u}_{i},{v}_{i},{t}_{i})$ is the local intercept surface, representing the baseline level at location $({u}_{i},{v}_{i})$ and time *t*; ${\beta }_{j}({u}_{i},{v}_{i},{t}_{i})$ is the spatiotemporally varying coefficient; and ${\epsilon }_{it}$ is the local spatiotemporal residual, capturing the remaining variation in ${Y}_{it}$ that cannot be explained by the local linear relationship at the given space–time coordinate.

#### Evaluation of the accuracy

Model performance was evaluated using the corrected Akaike information criterion (AICc), which balances model fit and complexity. A lower AICc value indicates a better model [33]. This model is expressed as [34–36]:

8 $$\mathrm{AICc}=-2\mathrm{ln}(L)+2k\cdot \frac{n}{n-k-1}$$,

where *n* is the sample size; *k* is the number of model parameters; and *L* is the maximum likelihood of the model.

The coefficient of determination (*R*^2^) measures the proportion of variance in the dependent variable explained by the independent variables [37, 38]. The model is expressed as [39]:

9 $${R}^{2}=1-\frac{{\sum }_{i=1}^{n}{({y}_{i}-{\widehat{y}}_{i})}^{2}}{{\sum }_{i=1}^{n}{({y}_{i}-\overline{y })}^{2}}$$,

where ${y}_{i}$ denotes the observed value, ${\widehat{y}}_{i}$ represents the predicted value; and $\overline{y }$ represents the sample mean.

Adjusted *R*^2^ modifies *R*^2^ to account for the number of predictors, avoiding the overestimation of model fit because of the inclusion of irrelevant variables [40]. The model is expressed as [39]:

10 $$\mathrm{Adjusted}{R}^{2}=1-(1-{R}^{2})\cdot \frac{n-1}{n-p-1}$$,

 where *n* is the sample size; *p* is the number of independent variables (features) in the model; and *n − p − *1 is the degrees of freedom.

## Results

### Spatiotemporal distribution and spatial clustering characteristics of CL

#### Global Moran’s I

Based on epidemiological data of CL for 1990–2021, in this study we used Global Moran's I to quantitatively assess the spatial distribution characteristics of the prevalence of and DALYs due to CL. This index identifies aggregation patterns by measuring the spatial dependence of the attribute values of geographical units.

The prevalence analysis revealed significant spatial heterogeneity. Moran's I was positive for most of the observed years and reached statistical significance (*Z* > 1.96, *P* < 0.05), indicating a continuous spatial positive correlation. This spatial dependence manifested as areas with a high prevalence tending to be spatially adjacent, whereas areas with a low prevalence exhibited spatial clustering, forming a significant spatial heterogeneity pattern. DALYs exhibited significant spatial heterogeneity; for > 60% of the observed years, Moran's I was positive and reached statistical significance (*Z* > 1.96, *P* < 0.05), and its spatial clustering pattern was relatively close to that of prevalence. Notably, time-series analysis of Moran’s I revealed a progressive weakening in the spatial clustering of prevalence and DALYs. This dynamic evolution of spatial distribution patterns may stem from the combined effect of multiple factors, including the effectiveness of public health interventions, refinement and adjustment of medical and health resource allocation, changes in regional environmental characteristics and shifts in socioeconomic conditions. This finding provides new empirical evidence for understanding the spatial evolution of CL (Table 2).

**Table 2:** Global Moran's I

Year	Prevalence			DALYs		
	Moran’s I	*Z-*score	*P*-value	Moran’s I	*Z-*score	*P*-value
1990	0.102	2.832	0.005	0.102	2.832	0.005
1991	0.101	2.810	0.005	0.101	2.811	0.005
1992	0.100	2.788	0.005	0.100	2.787	0.005
1993	0.099	2.766	0.006	0.099	2.767	0.006
1994	0.098	2.745	0.006	0.098	2.748	0.006
1995	0.098	2.728	0.006	0.097	2.728	0.006
1996	0.097	2.715	0.007	0.097	2.713	0.007
1997	0.097	2.704	0.007	0.097	2.703	0.007
1998	0.096	2.692	0.007	0.096	2.693	0.007
1999	0.096	2.675	0.007	0.096	2.676	0.007
2000	0.095	2.651	0.008	0.095	2.653	0.008
2001	0.093	2.604	0.009	0.093	2.606	0.009
2002	0.090	2.528	0.011	0.089	2.528	0.011
2003	0.086	2.437	0.015	0.086	2.436	0.015
2004	0.082	2.347	0.019	0.082	2.344	0.019
2005	0.079	2.275	0.023	0.079	2.270	0.023
2006	0.076	2.208	0.027	0.076	2.202	0.028
2007	0.073	2.123	0.034	0.072	2.117	0.034
2008	0.068	2.027	0.043	0.068	2.021	0.043
2009	0.064	1.927	0.054	0.063	1.920	0.055
2010	0.060	1.835	0.067	0.059	1.828	0.068
2011	0.055	1.733	0.083	0.055	1.726	0.084
2012	0.050	1.673	0.107	0.050	1.603	0.109
2013	0.045	1.487	0.137	0.044	1.475	0.140
2014	0.040	1.367	0.171	0.039	1.355	0.175
2015	0.035	1.265	0.206	0.035	1.251	0.211
2016	0.031	1.158	0.247	0.030	1.144	0.253
2017	0.026	1.050	0.294	0.256	1.037	0.300
2018	0.023	0.971	0.332	0.022	0.957	0.339
2019	0.022	0.940	0.347	0.021	0.926	0.355
2020	0.022	0.940	0.347	0.021	0.926	0.354
2021	0.047	1.595	0.111	0.021	0.926	0.354

DALYs, disability-adjusted life years.

#### Hot spot analysis

**Fig. 3:**
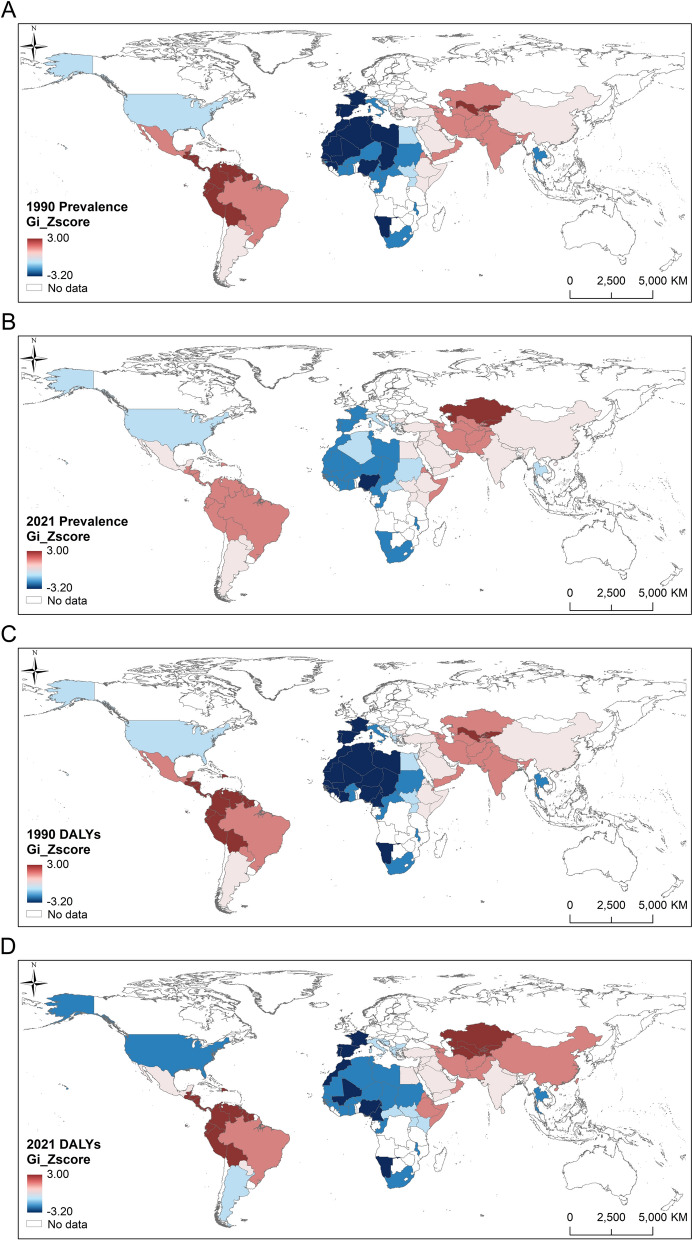
The hot and cold index of the prevalence of and DALYs due to  cutaneous leishmaniasis (CL) in 1990 and 2021. **A, B** The hot and cold index of the prevalence of CL in 1990 (**A**) and in 2021 (**B**). **C, D** The hot and cold index of DALYs due to CL in 1990 (**c**) and in 2021 (**D**). DALYs, disability-adjusted life years.

We obtained the Gi_Zscore values for all countries and regions within the study area through hot spot analysis (Getis–Ord Gi*), which measures the spatial clustering degree of the prevalence of and DALYs due to CL at a global scale. Gi_Zscore > 0 and Gi_Zscore < 0 indicated high and low spatial clustering, respectively. Figure 3 presents the hot and cold spots of the prevalence of and DALYs due to CL in 1990 and 2021. The major hot spot areas are shown to be in southwest Asia, including countries such as Kyrgyzstan, Saudi Arabia and Tajikistan, and in Central America, including countries such as Nicaragua, Costa Rica and Panama; cold spots are almost entirely concentrated in Africa, with sporadic occurrences in a few other countries, such as the USA and China.

The cold and hot spot distributions of DALYs and prevalence exhibited relatively similar patterns. The distribution of the centers of gravity of cold spots was concentrated mainly in Africa and southern European coastal countries (e.g. Spain, France and Italy). Hot spots exhibited the characteristic of “small clusters, wide dispersion.” One major hot spot was located in Panama and Colombia in Central America, and another was located in Uzbekistan, Kazakhstan and Kyrgyzstan in Central Asia.

#### Analysis of the moving trajectories of the centers of gravity

Using ArcGIS (ArcMap 10.8), we obtained the centers of gravity of hot and cold spots of prevalence and DALYs for 1990–2021. The moving trajectories were analyzed and standard deviation ellipses were plotted.

**Fig. 4:**
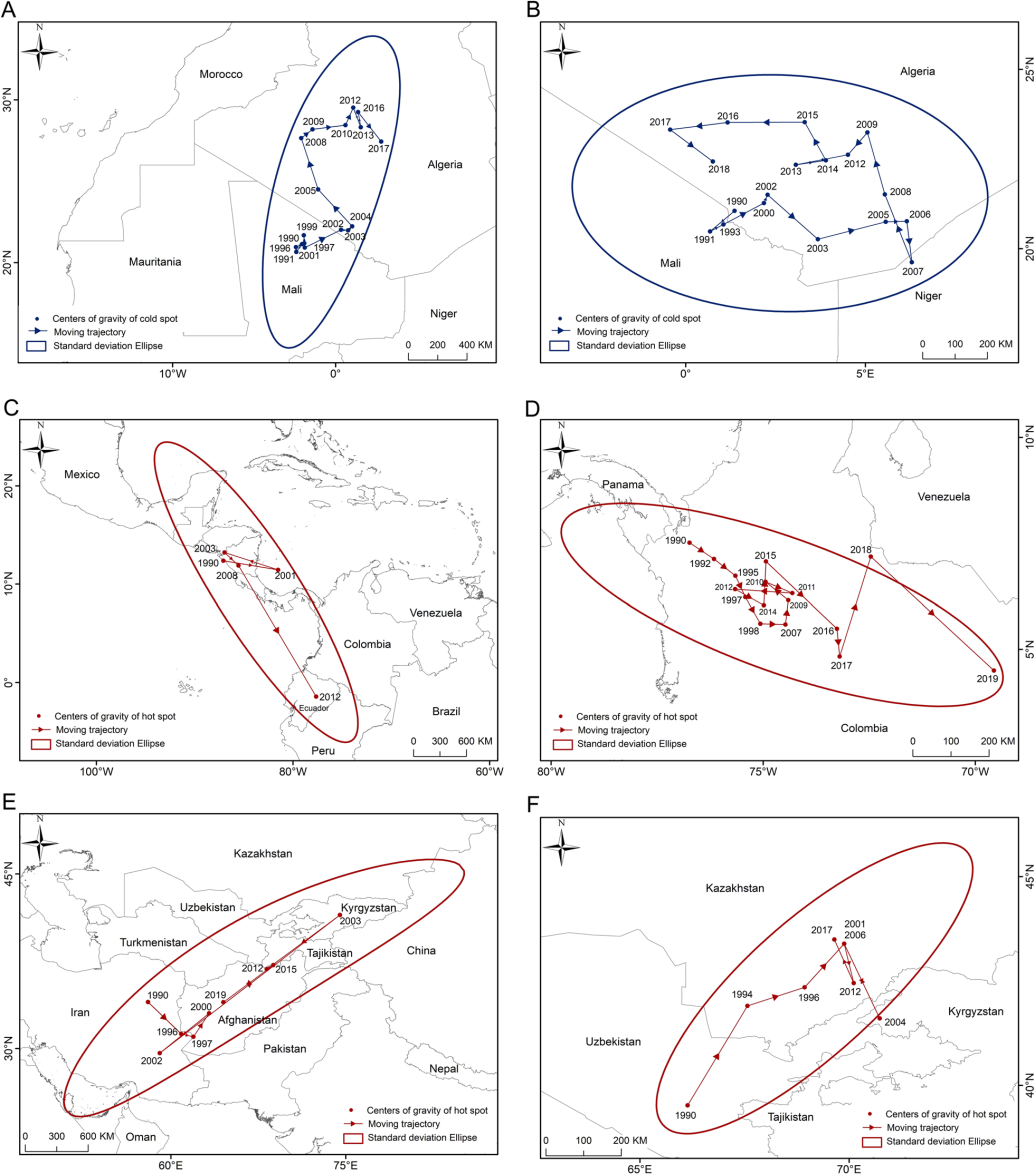
Moving trajectories of the centers of gravity of cold spots (**A** prevalence, **B** DALYs) and hot spots (**C**, **E** prevalence, **D**, **F** DALYs) of cutaneous leishmaniasis from 1990 to 2021. DALYs, disability-adjusted life years.

The centers of gravity of cold spots of prevalence were found to be concentrated together, mainly located within Mali and Algeria in North Africa, and showed overall northward migration (Fig. 4A). In contrast, the centers of gravity of the hot spots exhibited more significant spatiotemporal variation. Before 2014, the two hot spot areas were located in Central America and Central Asia. The centers of gravity of hot spots of prevalence in the Americas generally showed a migration from northwestern to southeastern (Fig. 4C), whereas those of hot spots of prevalence in Central Asia exhibited a southwest–northeast trend. After 2014, although Central America showed a relatively high prevalence of CL, hot spots in Central America disappeared, and only appeared in Central Asia. At this time, the centers of gravity of hot spots in Central Asia migrated to the southwest (Fig. 4E). Hot spots may disappear or migrate when epidemic determinants undergo substantial changes. Taking Central America as an example, the equatorial eastern Pacific entered a strong El Niño phase in 2014 that continues to this day [41], with significantly less precipitation in two consecutive rainy seasons. The consequence has been a significant drop in humidity in the forest–pastoral ecotone, where sand flies live; concurrently, forest fires caused by drought [42] further destroyed sand fly habitats. This may lead to a decrease in the prevalence of CL in Central America, where the center of gravity of the hot spot dissipated after 2014. However, further validation using finer-scale climate and vector data is needed [43].

The cold and hot spots of DALYs exhibited distinct spatiotemporal dynamic characteristics. However, compared to prevalence, the centers of gravity of cold and hot spots of DALYs migrated more frequently, but within a relatively smaller distance. The centers of gravity of the cold spots for DALYs were predominantly in Algeria, first moving eastward, then northward and finally westward (Fig. 4B). The centers of gravity of the hot spots for DALYs in the Americas were consistently located within Colombia, indicating that CL cases were concentrated in Colombia and its surrounding countries. The migration trajectory of the centers of gravity of the hot spots generally showed a northwest–southeast trend (Fig. 4D). Another hot spot was located in Central Asia, and its migration trajectory generally exhibited a southwest–northeast trend. Before 2005, the migration distance was large: moving from Uzbekistan across Kazakhstan to Kyrgyzstan, returning to Kazakhstan, and subsequently beginning to migrate within a small distance (Fig. 4F).

### Analysis of factors affecting the prevalence of and DALYs due to CL

#### The impact of environmental and socioeconomic factors on CL

**Fig. 5:**
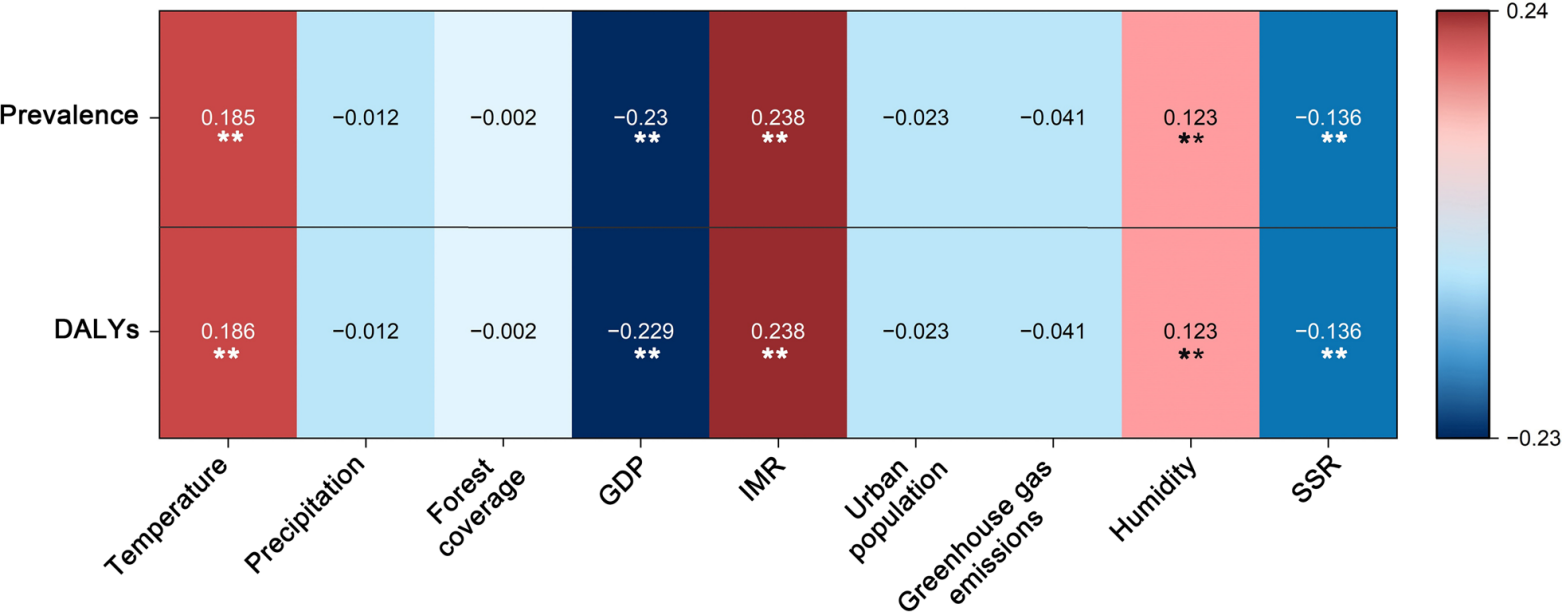
Correlation coefficients between each environmental and socioeconomic factor and the prevalence of and diability-adjusted life years due to cutaneous leishmaniasis. Asterisks indicate a statistically significant correlation with the denoted environmental and socioeconomic factors at **P < 0.01. GDP, gross domestic product; IMR, infant mortality rate; SSR, surface solar radiation.

The annual average values of all environmental and socioeconomic factors in 92 countries from 1990, 1995, 2000, 2005, 2010, 2015 and 2020 were obtained and normalized, and Spearman’s correlation analysis was used to determine their correlations with the normalized prevalence of and DALYs due to CL (Fig. 5).

The annual average temperature, IMR and humidity all showed significant positive correlations with the prevalence of and DALYs due to CL, with the correlation coefficient of IMR reaching 0.238, whereas GDP and SSR showed significant negative correlations with the prevalence of and DALYs due to CL, with the correlation coefficient of GDP reaching − 0.23. Additionally, annual precipitation, forest coverage, urban population and greenhouse gas emissions all showed negative correlations with the prevalence of and DALYs due to CL but did not reach significant levels.

In summary, the prevalence of and DALYs due to CL significantly correlated with environmental factors, including temperature, humidity, and SSR, and socioeconomic factors, including IMR and GDP.

#### Model results and accuracy evaluation

Based on the data collected on the prevalence of and DALYs due to CL from 92 countries, along with nine factors (temperature, precipitation, humidity, SSR, forest coverage, GDP, urban population, IMR, and greenhouse gas emissions), we established OLS, GWR and GTWR models at the global level. All data were first normalized. Taking spatial nonstationarity and differences in data density across continents into consideration, we also performed modeling at the continental level for prevalence and DALYs, and the results are shown in Tables 3 and 4. The GTWR model demonstrated the best regression performance for prevalence, with adjusted *R*^2^ values for prevalence of 0.841, 0.984, 0.839 and 0.972 (Table 3) and those for DALYs of 0.816, 0.966, 0.837 and 0.972 (Table 4), in Asia, Europe, the Americas and Africa, respectively. In the GWR model, the adjusted *R*^2^ for prevalence reached 0.775, 0.963, 0.788 and 0.963 and those for DALYs reached 0.773, 0.964, 0.786 and 0.963, in Asia, Europe, Americas and Africa, respectively. Both the GTWR and GWR models showed higher accuracy than the OLS model, in which the* R*^2^ for prevalence in the latter reached 0.387, 0.737, 0.302 and 0.295 and those for DALYs reached 0.385, 0.734, 0.300 and 0.296, in Asia, Europe, the Americas and Africa, respectively. This result is because the OLS model provides only a preliminary estimate of the linear relationship between the independent and dependent variables without considering complex nonlinear relationships. The GWR model considers spatial heterogeneity, and GTWR model further considers temporal heterogeneity based on GWR, thus demonstrating superior goodness-of-fit in this study.

**Table 3:** Model performance evaluation results for prevalence of cutaneous leishmaniasis

Region	Model	Residual square	Sigma	AICc	*R*^2^	Adjusted* R*^2^
Asia	OLS	3.092		− 237	0.387	
	GWR	1.087	0.074	− 272	0.786	0.775
	GTWR	0.770	0.063	− 248	0.848	0.841
Europe	OLS	< 0.001		− 1266	0.737	
	GWR	< 0.001	< 0.001	− 1249	0.967	0.963
	GTWR	< 0.001	< 0.001	− 1100	0.986	0.984
Americas	OLS	0.737		− 317	0.302	
	GWR	0.210	0.039	− 392	0.802	0.788
	GTWR	0.160	0.034	− 349	0.849	0.839
Africa	OLS	0.437		− 711	0.295	
	GWR	0.017	0.010	− 1183	0.965	0.963
	GTWR	0.017	0.009	− 117	0.973	0.972
Global	OLS	7.146		− 1051	0.060	
	GWR	3.894	0.078	− 1346	0.489	0.481
	GTWR	3.752	0.076	− 128	0.507	0.500

AICc , Akaike information criterion corrected; OLS, ordinary least squares; GWR, geographically weighted regression; GTWR, geographically and temporally weighted regression;* R*^*2*^, coefficient of determination.

**Table 4:** Model performance evaluation results for disability-adjusted life years due to cutaneous leishmaniasis

Region	Model	Residual square	Sigma	AICc	*R*^2^	Adjusted* R*^2^
Asia	OLS	3.068		− 239	0.385	
	GWR	1.085	0.074	− 272	0.783	0.773
	GTWR	0.879	0.067	− 270	0.825	0.816
Europe	OLS	< 0.001		− 1261	0.734	
	GWR	< 0.001	< 0.001	− 1248	0.968	0.964
	GTWR	< 0.001	< 0.001	− 1187	0.970	0.966
Americas	OLS	0.733		− 318	0.300	
	GWR	0.211	0.039	− 391	0.800	0.786
	GTWR	0.161	0.0339	− 350	0.847	0.837
Africa	OLS	0.427		− 716	0.296	
	GWR	0.022	0.010	− 1186	0.965	0.963
	GTWR	0.016	0.009	− 1178	0.973	0.972
Global	OLS	7.058		− 1059	0.060	
	GWR	3.864	0.077	− 1350	0.486	0.479
	GTWR	3.724	0.076	− 1290	0.505	0.498

AICc , Akaike information criterion corrected; OLS, ordinary least squares; GWR, geographically weighted regression; GTWR, geographically and temporally weighted regression;* R*^*2*^, coefficient of determination.

This study simultaneously conducted modeling of the prevalence and DALYs in 92 countries with CL at the global level, and GTWR also showed a higher goodness-of-fit than GWR and OLS (Tables 3, 4). All GTWR models at the continental level had higher adjusted *R*^2^ values than the GTWR model at the global level, which may be attributed to scale differences in spatial heterogeneity. GTWR assumes “the closer, the more similar.” However, when the study area expands to a global scale, this assumption is severely undermined by various factors, and modeling by continent mitigates these disruptions. This can also be verified by the bandwidth of each GTWR model; the GTWR controls the spatial weight decay through the bandwidth. On the global scale, disease transmission is influenced by intercontinental differences, resulting in multimodal heterogeneity. For example, the GTWR bandwidths for prevalence in Asia, Europe, the Americas and Africa were 0.118314, 0.119944, 0.114996 and 0.114996, respectively. In contrast, the global GTWR bandwidth uses a compromised bandwidth (bandwidth = 0.112654), leading to a distortion in the weight distribution and a decrease in the goodness-of-fit.

#### Coefficients of explanatory variables in the GTWR model

To further clarify the independent effects of environmental and socioeconomic factors on the prevalence of and DALYs due to CL, we first conducted multicollinearity diagnostics for all independent variables. As shown in Table 5, the variance inflation factors (VIF) of all factors were < 7.1, indicating that no multicollinearity was detected according to the commonly used threshold of 10 [44]. In addition, the GTWR model, which exhibited the best-fitting accuracy, alleviates multicollinearity through mechanisms such as adaptive bandwidth selection, which in turn optimizes the local neighborhood size to maximize parameter variability, and a spatiotemporal weighting scheme, which increases local sample heterogeneity through geographical and temporal proximity weighting [45, 46]. These characteristics naturally mitigate potential collinearity issues in local coefficient estimations. The GTWR model was employed as a benchmark to extract and summarize the standardized regression coefficients of each environmental and socioeconomic factor. Based on these results, the individual contributions of each factor to the prevalence of and DALYs due to CL were evaluated.

**Table 5:** Results of the collinearity diagnostic analysis of environmental and socioeconomic factors

Factor	Tolerance	Variance inflation factors
Urban population	0.456	2.191
Humidity	0.141	7.070
Forest coverage	0.298	3.359
GDP	0.735	1.360
Precipitation	0.152	6.596
IMR	0.450	2.224
Temperature	0.333	3.003
SSR	0.982	1.018
Greenhouse gas emissions	0.927	1.079

GDP, gross domestic product; IMR, infant mortality rate; SSR, surface solar radiation.

The environmental and socioeconomic factors that dominated the prevalence of CL on each continent showed significant spatial heterogeneity (Table 6). In Africa, greenhouse gas emissions were the dominant factor (mean 0.546, standard deviation [SD] 2.268), exhibiting a stable positive effect, whereas the absolute values of the coefficients of the remaining variables were all < 0.15, indicating a limited contribution. In the Americas, the greenhouse gas emissions showed a strong negative correlation (mean − 2.431, SD 2.159) with the greatest variability. Urban population, temperature and IMR all exerted positive influences (mean 0.241, 0.231 and 0.207, respectively); however, their absolute values were < 1/10 that of greenhouse gas emissions, indicating a relatively weaker contribution. In Europe, the absolute values of the coefficients of all factors were < 0.020, and the SD were ≤ 0.030, suggesting that the marginal effects of these environmental and socioeconomic factors on the prevalence of CL are generally weak. In Asia, urban population, humidity and forest coverage constituted the dominant negative factors (mean − 0.801, − 0.714 and − 0.702, respectively); GDP and precipitation exhibited significant positive effects (mean 0.689 and 0.480, respectively). The mean of the coefficients of greenhouse gas emissions was close to zero; however, the standard deviation reached 2.06, indicating that its effect varied spatially and exhibited high heterogeneity.

**Table 6:** Coefficients of each factor in the GTWR model for prevalence across continents

Region	Factor	Mean	Standard deviation	Maximum	Minimum
Asia	Urban population	− 0.801	0.532	0.232	− 2.365
	Humidity	− 0.714	0.628	0.357	− 3.066
	Forest coverage	− 0.702	0.640	0.757	− 2.023
	GDP	0.689	0.982	5.370	− 1.793
	Precipitation	0.480	0.723	1.822	− 2.506
	IMR	− 0.415	0.464	0.550	− 1.497
	Temperature	0.304	0.373	1.955	− 0.236
	SSR	− 0.188	0.177	0.347	− 0.678
	Greenhouse gas emissions	0.097	2.062	5.236	− 6.268
Europe	Greenhouse gas emissions	− 0.013	0.030	0.035	− 0.095
	IMR	0.005	0.006	0.031	− 0.006
	GDP	− 0.003	0.004	0.003	− 0.015
	Temperature	− 0.002	0.004	0.008	− 0.016
	Forest coverage	− 0.001	0.002	0.003	− 0.010
	Humidity	Abs < 0.001	0.002	0.004	− 0.007
	Urban population	Abs < 0.001	0.001	0.005	− 0.002
	SSR	Abs < 0.001	0.001	0.002	− 0.003
	Precipitation	Abs < 0.001	0.001	0.003	− 0.003
Americas	Greenhouse gas emissions	− 2.431	2.159	3.212	− 8.154
	Urban population	0.241	0.166	0.541	− 0.232
	Temperature	0.231	0.388	1.092	− 0.430
	IMR	0.207	0.261	0.543	− 0.949
	Forest coverage	0.138	0.241	0.697	− 0.285
	GDP	− 0.070	0.753	1.979	− 1.755
	Humidity	− 0.028	0.268	0.782	− 0.612
	SSR	− 0.025	0.089	0.114	− 0.409
	Precipitation	0.012	0.156	0.272	− 0.339
Africa	Greenhouse gas emissions	0.546	2.268	6.012	− 5.151
	Precipitation	− 0.138	0.332	0.238	− 1.458
	Forest coverage	0.058	0.403	1.605	− 1.263
	GDP	0.058	1.042	2.743	− 3.497
	Temperature	0.049	0.137	0.567	− 0.223
	IMR	− 0.022	0.089	0.260	− 0.350
	Urban population	0.013	0.141	0.573	− 0.214
	Humidity	− 0.010	0.225	0.583	− 0.555
	SSR	0.008	0.022	0.115	− 0.033

Abs , absolute value; GDP, gross domestic product; IMR, infant mortality rate; SSR, surface solar radiation.

Greenhouse gas emissions were the dominant positive and negative variables in Africa and the Americas, respectively, whereas urbanization and environmental factors were dominant in Asia, and the effect of each variable was relatively weak in Europe. Therefore, the key risk factors need to be considered differently for each continent.

**Fig. 6:**
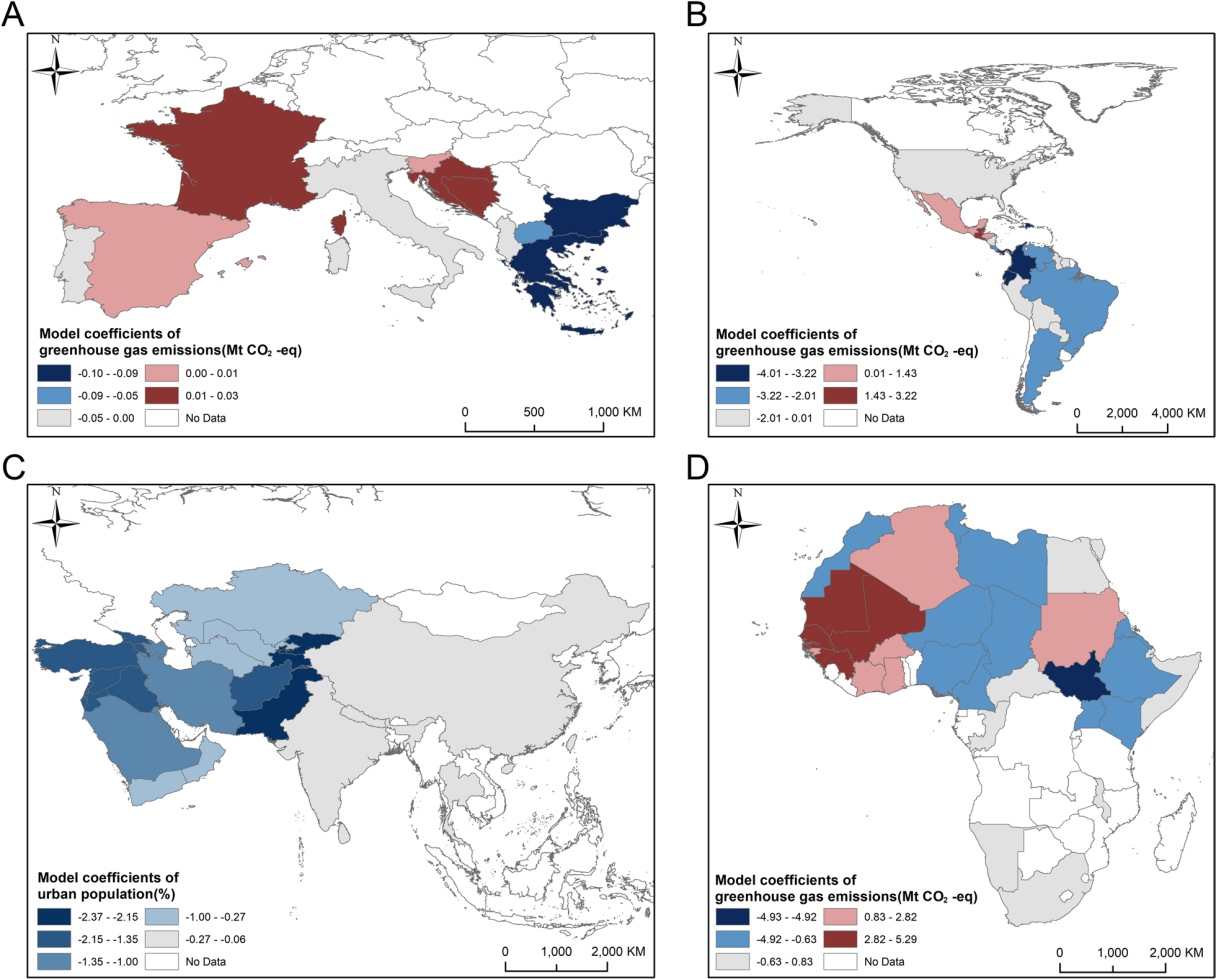
Distribution of coefficients of dominant factors for prevalence across continents in the geographically and temporally weighted regression model.** A, B, D **Coefficients of greenhouse gas emissions in Europe (**A**), the Americas (**B**) and Africa (**D**). **C** Coefficients of urban population in Asia

Figure 6 shows the coefficients of the dominant factors influencing prevalence within each continent. In Africa, coefficients of greenhouse gas emissions exhibited large differences in values (maximum [max] 5.2837, minimum [min] − 4.9235). The maximum and minimum values appeared in northwestern Africa and Central Africa, respectively (Fig. 6D). In the Americas, coefficients of greenhouse gas emissions ranging from − 3.22 to − 2.01 were widely distributed in southern North America and eastern South America, with local extreme values of less than − 3.22, whereas positive coefficients only sporadically appeared in southeastern North America, indicating that the contribution of greenhouse gas emissions had high spatial stability in the Americas (Fig. 6B). In Europe, the marginal effect of greenhouse gas emissions on the prevalence of CL was weak. The negative coefficients were mainly located in Eastern Europe, and the positive coefficients were located in Western and Central Europe (Fig. 6A). In Asia, the dominant factor was urban population. The coefficients in most areas of West and Central Asia were between − 2.37 and − 0.27, but showed a weak negative impact in East Asia and Southeast Asia (Fig. 6C).

The marginal impact of each factor on DALYs due to CL across continents was measured (Table 7). The spatial influence of the coefficients was similar to that of prevalence. In Africa, greenhouse gas emissions remained the only strong driving factor (mean 0.540, SD 2.253), whereas in the Americas, the effect of greenhouse gas emissions was opposite to that in Africa (mean − 2.416, SD 2.147). Although the proportion of urban population, temperature and IMR showed a positive effect (mean 0.240, 0.227, and 0.203, respectively), their absolute values were < 1/10 of those of greenhouse gas emissions. In Europe, the absolute values of coefficients for all environmental and socioeconomic factors were ≤ 0.012, with standard deviations of < 0.021 and ranges of < 0.32. In Asia, urban population was the most important explanatory factor (mean − 0.774, SD 0.483), followed by forest coverage and humidity (mean − 0.703 and − 0.655, respectively). In contrast, GDP and precipitation showed a positive effect (mean 0.659 and 0.469, respectively).

**Table 7:** Coefficients of each factor in the geographically and temporally weighted regression model for disability-adjusted life years across continents

Region	Factor	Mean	Standard deviation	Maximum	Minimum
Asia	Urban population	− 0.774	0.483	0.094	− 1.914
	Forest coverage	− 0.703	0.598	0.481	− 1.901
	Humidity	− 0.655	0.581	0.378	− 2.656
	GDP	0.595	0.828	4.086	− 1.242
	Precipitation	0.469	0.602	1.404	− 1.588
	IMR	− 0.406	0.444	0.401	− 1.133
	Temperature	0.293	0.334	1.613	− 0.238
	SSR	− 0.179	0.159	0.109	− 0.585
	Greenhouse gas emissions	− 0.013	1.763	3.927	− 7.922
Europe	Greenhouse gas emissions	− 0.012	0.021	0.015	− 0.086
	IMR	0.006	0.006	0.030	− 0.002
	GDP	− 0.002	0.003	0.003	− 0.012
	Temperature	− 0.002	0.003	0.006	− 0.011
	Forest coverage	− 0.001	0.002	0.003	− 0.005
	Urban population	Abs < 0.001	0.001	0.005	− 0.001
	Humidity	Abs < 0.001	0.002	0.002	− 0.006
	SSR	Abs < 0.001	0.001	0.001	− 0.003
	Precipitation	Abs < 0.001	0.001	0.002	− 0.003
Americas	Greenhouse gas emissions	− 2.416	2.147	3.217	− 8.131
	Urban population	0.240	0.164	0.543	− 0.226
	Temperature	0.227	0.389	1.087	− 0.429
	IMR	0.203	0.258	0.531	− 0.938
	Forest coverage	0.138	0.239	0.693	− 0.280
	GDP	− 0.070	0.740	1.964	− 1.703
	Humidity	− 0.025	0.267	0.777	− 0.605
	SSR	− 0.023	0.088	0.115	− 0.407
	Precipitation	0.011	0.156	0.270	− 0.337
Africa	Greenhouse gas emissions	0.540	2.253	5.968	− 5.089
	Precipitation	− 0.136	0.327	0.238	− 1.437
	GDP	0.059	1.034	2.743	− 3.441
	Forest coverage	0.056	0.399	1.586	− 1.262
	Temperature	0.048	0.136	0.558	− 0.226
	IMR	− 0.022	0.088	0.258	− 0.350
	Urban population	0.013	0.140	0.570	− 0.213
	Humidity	− 0.009	0.223	0.579	− 0.548
	SSR	0.008	0.022	0.115	− 0.033

Abs , absolute value; GDP, gross domestic product; IMR, infant mortality rate; SSR, surface solar radiation.

**Fig. 7:**
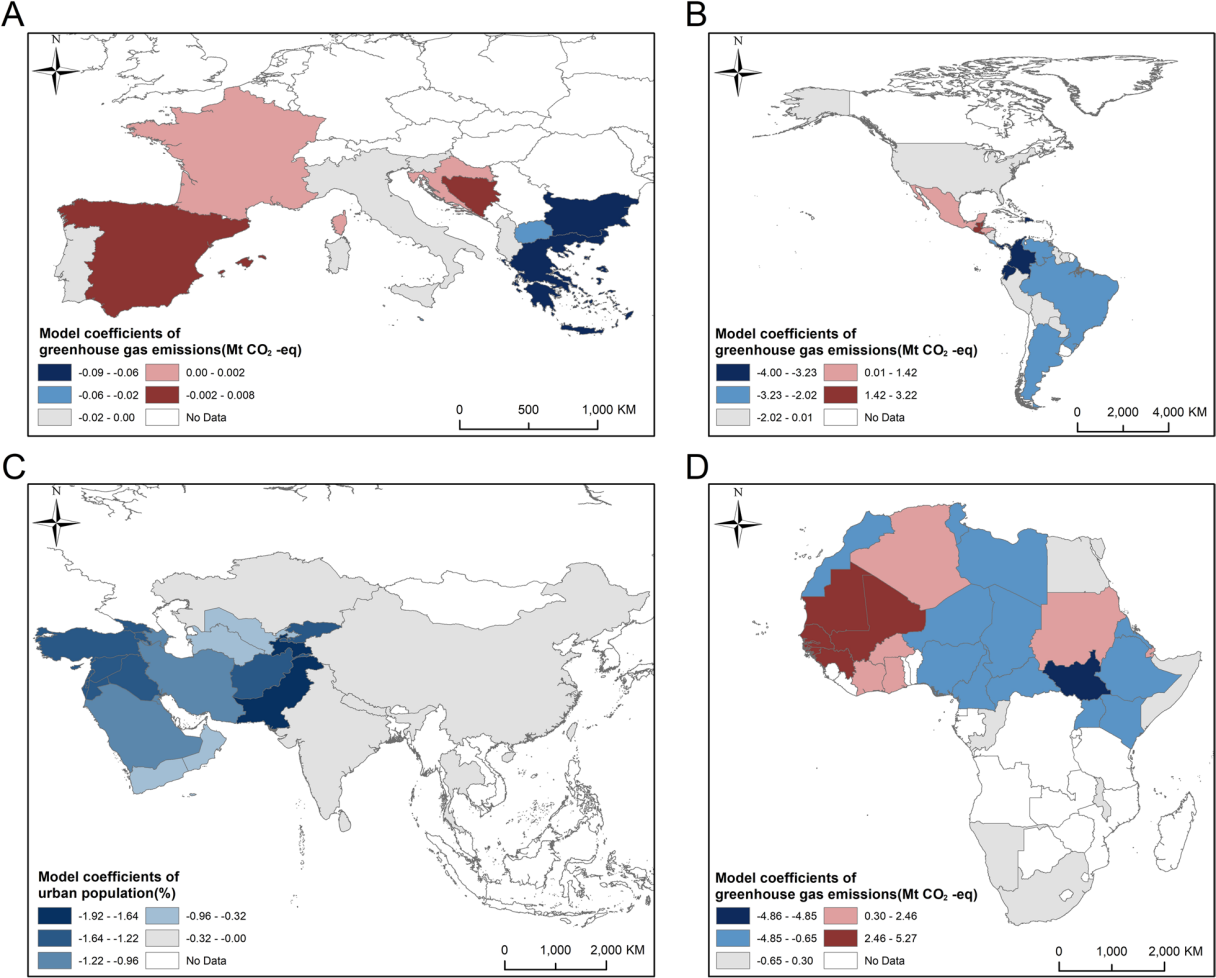
Distribution of coefficients of dominant factors for disability-adjusted life years across continents in the geographically and temporally weighted regression model. ** A, B, D **Coefficients of greenhouse gas emissions in Europe (**A**), the Americas (**B**) and Africa (**D**). **C** Coefficients of urban population in Asia

The distribution of the coefficients of the dominant influencing factors in DALYs within each continent revealed high consistency with their distribution in prevalence (Fig. 7). In Africa, coefficients for greenhouse gas emissions ranged from a maximum of 5.2616 to a minimum of − 4.8528, indicating its large spatial fluctuations within the continent (Fig. 7D). In the Americas, the negative coefficients of the greenhouse gas emissions were widely distributed, whereas positive coefficients appeared sporadically only in southern North America (Fig. 7B). The marginal effect of greenhouse gas emissions in Europe remained similar to that of prevalence (Fig. 7A). In Asia, the effect of urban population was dominant, and negative coefficients were mainly concentrated in West and Central Asia (Fig. 7C).

## Discussion

This study focused on the prevalence of and DALYs due to CL in 92 countries. Spatial analysis methods, such as spatial autocorrelation (Moran’s I) and directional distribution (standard deviation ellipse), were used to systematically analyze the distribution characteristics of the prevalence of and DALYs due to CL. Spearman’s correlation analysis was conducted to identify influencing factors. By integrating RS-retrieved data and GIS technologies, we established GTWR, GWR and OLS models to systematically explore the effects of environmental and socioeconomic factors, providing an explanatory basis for the prevalence of and DALYs due to CL.

Regarding Global Moran’s I, we observed a continuous decline in prevalence and DALYs. Significant clustering (*P* < 0.05) was observed before 2008; however, this significance disappeared in the years after 2008. Before 2008, CL cases were largely distributed in Latin America and Central Asia, consistent with reports provided by the WHO [3]. These regions have stable and favorable conditions for the survival and reproduction of sand flies, characterized by poor sanitation practices and systems [47, 48], poor housing conditions [49–51] and a relatively large malnourished population [52]. In addition, global population movements were relatively few between 1990 and 2008, and disease transmission was mainly limited to small regions, forming “hot spot clusters.” Since the late 2000 s, conflicts, such as the Middle East wars, Venezuelan migration crisis and Afghan conflict have erupted, triggering sharp surges in localized population movements (for example, the proportion of Syrian refugees and asylum-seekers increased from 2.41% in 2005 to 33.73% in 2020) [53–58]. These conflicting hot spots coincide precisely with the case clusters identified in the present study (Central Asia and Central America); population movement may transport cases from traditional high-epidemic regions to non-endemic areas, such as Yemen and Afghanistan. Consequently, cases are no longer confined to a small number of countries or regions but are now widely distributed across many states, which may result in a marked decline in spatial clustering.

Second, in terms of environmental factors, temperature and humidity were significantly positively correlated and SSR was significantly negatively correlated with the prevalence of and DALYs due to CL. These findings can be explained by the biological living requirements of sand fly vectors. Within an optimal range, elevated temperatures accelerate the life and gonotrophic cycles of sand flies, thereby facilitating population growth. For example, laboratory data indicate that the intrinsic rate of population increase for *Phlebotomus papatasi* increases with temperature, peaking at 28 °C, and the developmental time from egg to adult is the shortest at 32 °C [59]. Similarly, the optimal oviposition temperature for *Lutzomyia longipalpis* increases with temperature, peaking between 25 °C and 26 °C [60]. Furthermore, humidity is crucial for the survival of sand flies. Queiroz et al. monitored and calculated the relative frequency and richness of sand flies in three districts in Brazil and found that *L. longipalpis* showed occurrence peaks during the rainy season and that there was a temporal correlation with humidity [61]. Conversely, the negative correlation of prevalence and DALYs with SSR is consistent with the nocturnal or crepuscular habits of most sand fly species [62]; sand fly larvae must inhabit environments rich in organic matter, moisture and shade, as direct exposure to SSR can result in mortality [63]. This is consistent with the results of the present study. However, the impacts of extreme weather events must also be considered. For example, anomalies in rainfall and temperature induced by the 2015–2016 El Niño episode produced changes in the potential distribution of CL vectors in Colombia, and the distribution of three species of sand flies (*Lutzomyia gomezi*, *Lutzomyia ovallesi*, and *Lutzomyia panamensis*) expanded, thereby increasing the number of cases in Columbia [64].

Additionally, we found that social factors (such as GDP and IMR) had a prominent impact on CL. GDP was significantly negatively correlated and IMR was significantly positively correlated with the prevalence of and DALYs due to CL. This finding may reflect the disparity in healthcare investments between developed and developing countries. A higher economic status (high GDP) typically implies greater financial resources allocated to the (public) health infrastructure, vector control programs and advanced medical treatments. Conversely, the IMR serves as a potent indicator of a nation’s overall healthcare standards [65, 66]. Consequently, elevated economic and healthcare levels may contribute to a reduction in the burden of CL. However, in addition to GDP and IMR, this study did not consider other factors, such as national policies regarding leishmaniasis, hygiene conditions and medical costs. For example, Costa Rica is a hot spot country, and its inflation rate fluctuates between 0.37% and 1.24%. Drug prices remain high due to market monopolies; for example, drug prices are high, and price increases were ranked among the highest in the world for 2 consecutive years (2008 and 2009) [67]. The Organization for Economic Co-operation and Development reported that a lack of competition in the pharmaceutical industry and treatment delays among low-income groups indirectly contribute to the increase in chronic cases [68, 69]. Using Morocco as another example, from 2010 to 2012, Morocco distributed strychnine-poisoned wheat bait in Errachidia Province [70] to kill sand rats, directly reducing the vector transmission of CL. In addition, the Moroccan government considers tourism to be a pillar industry and implements special subsidies for environmental sanitation and vector control in tourist hot spots, such as Marrakech and Fez, which protects the health of tourists and indirectly reduces the risk of infection for local residents. Thus, prevention and control policies for CL can alter its distribution.

In recent years, research has employed GIS as a tool for studying parasitic diseases. Wang et al. [71] and Liu et al. [72] compared the environmental driving factors affecting the distribution of *Oncomelania hupensis* in the Yangtze River Basin and Anhui, respectively, using GTWR, GWR and OLS. These authors showed that GTWR was the best model. Zhu et al. [73] used GTWR to analyze the influence of meteorological factors (temperature, humidity, precipitation and wind speed) on imported cases of dengue fever, with their results showing that GTWR (*R*^2^ = 0.20, AICc 1987.98) was superior to OLS (*R*^2^ = 0.15, AICc 1990.14). In another study, the GTWR model was compared with other models, such as the multinomial logistic regression (MLR) model [74]. For example, Yao et al. [75] explored the impact of climatic factors, such as wind speed, wind direction, relative humidity, air temperature and precipitation, on mortality due to malaria in sub-Saharan Africa. Their results showed that the GTWR model (*R*^2^ = 0.71) was superior to the MLR model (*R*^2^ = 0.14), confirming the association between malaria burden and geographical changes. In our study, the GTWR model also demonstrated the best regression performance, with adjusted* R*^2^ values for prevalence reaching 0.841, 0.984, 0.839 and 0.972 and those for DALYs reaching 0.816, 0.966, 0.837, and 0.972, in Asia, Europe, America, and Africa, respectively. Based on the above studies, we conclude that the GTWR has advantages in analyzing the spatiotemporal heterogeneity of parasitic diseases.

In future studies, additional possible potential factors (socioeconomic factors, such as medical care levels and medical costs, and environmental factors, such as land use and land cover) could be incorporated to improve the accuracy and explanatory power of the models, providing a useful reference for the analysis of global CL.

## Conclusions

To our knowledge, this study is the first to explore the spatiotemporal distribution patterns of the prevalence of and DALYs due to CL and quantitatively analyze the spatiotemporal effects of environmental and socioeconomic factors on CL on a global scale. Spearman’s correlation analysis revealed that temperature, IMR and humidity are positively correlated with the prevalence of and DALYs due to CL, whereas GDP and SSR are negatively correlated. Comparative modeling using OLS, GWR and GTWR models showed that GTWR achieved the highest *R*^2^, corroborating its superior capacity to accommodate spatiotemporal heterogeneity and capture the annual fluctuation in explanatory variables underlying the prevalence of and DALYs due to CL.

## Data Availability

The datasets supporting the conclusions of this article are listed in Table 1.
